# Whole genome SNP-associated signatures of local adaptation in honeybees of the Iberian Peninsula

**DOI:** 10.1038/s41598-018-29469-5

**Published:** 2018-07-24

**Authors:** Dora Henriques, Andreas Wallberg, Julio Chávez-Galarza, J. Spencer Johnston, Matthew T. Webster, M. Alice Pinto

**Affiliations:** 1 Mountain Research Centre (CIMO), Polytechnic Institute of Bragança, Campus de Sta. Apolónia, 5300-253 Bragança, Portugal; 2 Centre of Molecular and Environmental Biology (CBMA), University of Minho, Campus de Gualtar, 4710-057 Braga, Portugal; 3 Department of Medical Biochemistry and Microbiology, Science for Life Laboratory, Uppsala University, SE -751 23 Uppsala, Sweden; 4 Instituto Nacional de Innovación Agraria (INIA), Av. La Molina 1981, La Molina, Lima, Peru; 5 Department of Entomology, Texas A&M University, College Station, TX 77843-2475 USA

## Abstract

The availability of powerful high-throughput genomic tools, combined with genome scans, has helped identifying genes and genetic changes responsible for environmental adaptation in many organisms, including the honeybee. Here, we resequenced 87 whole genomes of the honeybee native to Iberia and used conceptually different selection methods (Samβada, LFMM, PCAdapt, iHs) together with *in sillico* protein modelling to search for selection footprints along environmental gradients. We found 670 outlier SNPs, most of which associated with precipitation, longitude and latitude. Over 88.7% SNPs laid outside exons and there was a significant enrichment in regions adjacent to exons and UTRs. Enrichment was also detected in exonic regions. Furthermore, *in silico* protein modelling suggests that several non-synonymous SNPs are likely direct targets of selection, as they lead to amino acid replacements in functionally important sites of proteins. We identified genomic signatures of local adaptation in 140 genes, many of which are putatively implicated in fitness-related functions such as reproduction, immunity, olfaction, lipid biosynthesis and circadian clock. Our genome scan suggests that local adaptation in the Iberian honeybee involves variations in regions that might alter patterns of gene expression and in protein-coding genes, which are promising candidates to underpin adaptive change in the honeybee.

## Introduction

In the current context of a global human-mediated environmental crisis, the long-standing goal of uncovering the genetic basis of adaptation has never been so important. Recent technological advances allow for major steps towards that goal. Increasingly powerful high-throughput sequencing and computational technologies, coupled with increasingly sophisticated analytical tools, have changed the scale of analysis from limited genomic regions and few loci to whole genomes, allowing thereby detection of signatures of selection at an unprecedented resolution and depth.

Most genome-wide analytical tools detect selection by searching for unusual patterns of genetic variation positing that population demographic history affects variation across all loci while natural selection operates at specific loci^1–4^. Known as outlier tests, selection footprints are sought by scanning genomes using a population-based differentiation measure such as F_ST_^5,6^ or by an individual-based approach centred on Bayesian factor models^7^. Another class of increasingly popular analytical tools, known as genetic-environment association (GEA) methods, identify selection by finding strong associations between genetic and environmental data^8–12^. By uncovering loci that are directly or indirectly correlated with the environmental factors, GEA methods can potentially identify selective pressures driving local adaptation^13–15^. A drawback of both classes of tools is that demographic processes and complex spatial structuring may create patterns resembling selection, leading to false positives^16–18^. However, a recently developed approach controls for population structure using latent factors estimated considering the statistical model and the data simultaneously. This approach has been incorporated into some outlier tests (e. g. the Bayesian factor model of PCAdapt^7^) and GEA methods (e. g. latent factor mixed model, LFMM^9^).

Studies using whole-genome scans have employed these analytical tools to identify hundreds of regions under selection in many model and non-model organisms^19–29^. This study further contributes to the rapidly growing list of organisms by helping uncover genetic pathways underlying local adaptation of one of the most diverse and evolutionarily complex honeybee subspecies, the Iberian honeybee (hereafter IHB), *Apis mellifera iberiensis*.

The honeybee (*Apis mellifera* L.) evolved into 31 currently recognized subspecies^30–34^, which have been grouped into four main evolutionary lineages: Northern and Western European, M; Southeastern European, C; African, A; and Middle Eastern, O^30^. In this wide range of diversity, the M-lineage IHB is one of the most intriguing subspecies, exhibiting complex patterns of clinal variation as have many other organisms that evolved in the Iberian glacial refuge (reviewed by Weiss and Ferrand^35^). Genetic surveys of the IHB have suggested that while evolutionarily neutral processes have played an important role in shaping the sharp northeastern-southwestern Iberian cline^36–40^, selection is a force that cannot be ignored^41^. Iberia possesses high physiographic complexity, with several large mountain ranges, and due to its geographical position is under the influence of both the North Atlantic and the Mediterranean seas. These features have shaped a diverse array of climates (including desert, Mediterranean, Alpine, and Atlantic) and plant communities with variable flowering peaks to which the IHB had to adapt.

A previous selection scan of the IHB using an array of 383 SNPs (single nucleotide polymorphisms) identified 34 putatively adaptive SNPs located in genes involved in vision, xenobiotic detoxification, and innate immune response^41^. However, the 383 SNPs were widely spaced, and given the unusually high recombination rate in honeybees^42^ genomic regions important in local adaptation have certainly been missed, as suggested by whole-genome studies of other subspecies^33,43–47^. In the present study, we employed a combination of outlier and GEA methods to identify genome-wide signatures of selection from 87 whole-genome sequences, thereby expanding the SNP-array scan of Chávez-Galarza, *et al*.^41^ by over 3 orders of magnitude (3367 fold). We approached local adaption in the IHB by addressing the following questions: Does adaptation arise from mutations that change amino acids? Which genes are responsible for adaptation to different environments? Which environmental factors might act as selective pressures in IHBs? In answering these questions, major insights will be gained toward understanding the genetic pathways used by the IHB to adapt to the broad range of Iberian environments.

## Results

**Figure 1:**
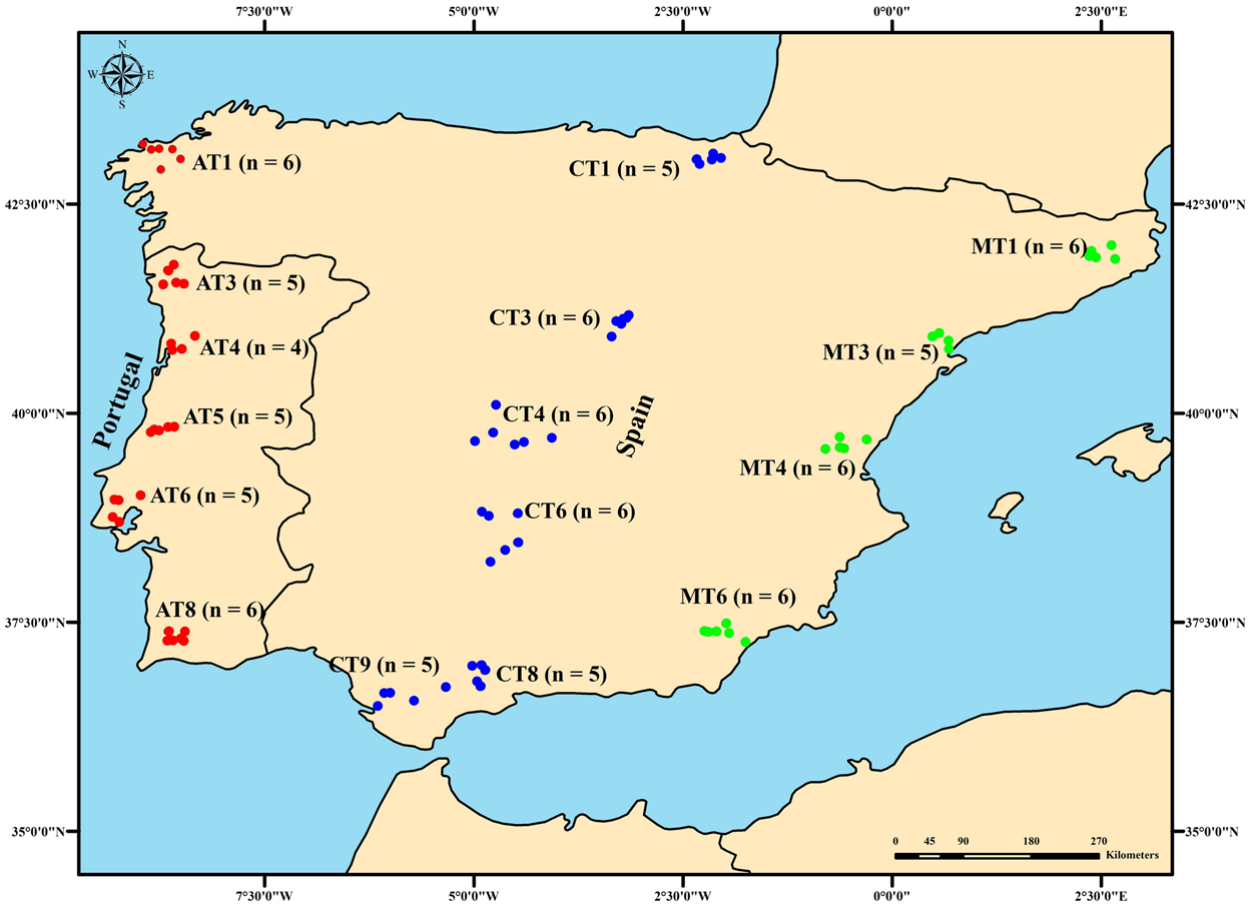
Location of sampling sites. The samples were distributed across three transects in the Iberian Peninsula, represented by three different colours: Atlantic in red (AT; N = 31), Central in blue (CT; N = 33), and Mediterranean in green (MT, N = 23). Each dot represents a single colony and apiary. Sampling site codes (AT1 to MT6) correspond to those reported by Chávez-Galarza, *et al*.^41^.

A total of 1,289,449 SNPs were retained, after the filtering process and using a minor allele frequency (MAF) > 0.05, for 87 resequenced IHBs sampled from across the Iberian range (Fig. 1). Of these, 670,738 SNPs were located in intergenic regions (120,301 in intergenic regions <2 Kb of exons, 37,058 in intergenic regions <1 Kb upstream of exons), 557,334 in introns (23,092 < 50 bp of exons), 18,841in UTRs (untranslated region), and 42,536 in exons (Supplementary Table S1). The average physical distance between SNPs was 170.262 bp varying between 1 bp and 136,266 bp (Supplementary Fig. S1).

### Population Structure

**Figure 2:**
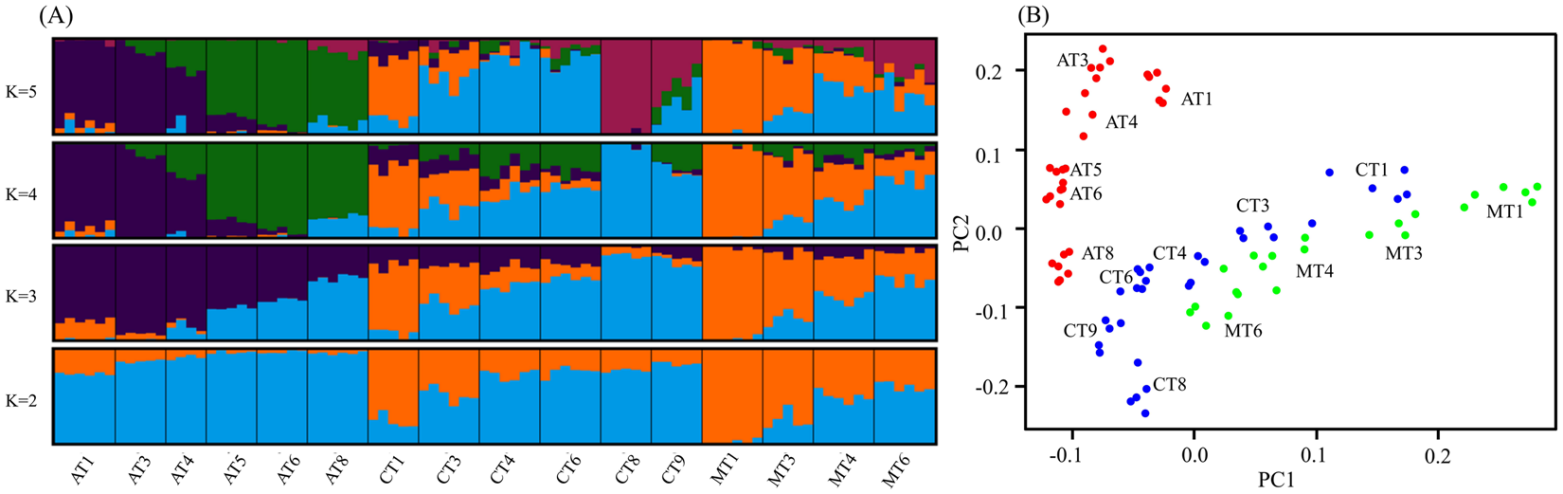
Population structure of *A. m. iberiensis*. (**A**) Structure estimated by sNMF from K = 2 to K = 5. The 16 sampling sites are arranged from north (AT1, CT1, MT1) to south (AT8, CT9, MT6) in each of the three transects. Plots represent each of the 87 individuals by a vertical bar partitioned into coloured segments (clusters) corresponding to membership proportions (Y-axis: 0-1) in each cluster. Vertical black lines separate the 16 sampling sites. (**B**) Score plot displaying the latent factors of each individual honeybee in PC1 and PC2 for K = 2. Each colour represents a different transect.

Population structure and demographic history can create genomic patterns that mimic selection. Accordingly, population structure was analysed to prevent discovery of false positives^16–18,48^. The genetic structure was inferred from the 1,289,449 SNPs with sNMF and PCAdapt, which identified one and two optimal number of clusters (K), respectively (Supplementary Fig. S2). Incongruent optimal K values can be obtained by different methods^49^, especially in the presence of low levels of population differentiation^50^, which is the case of the IHB with a global F_ST_ = 0.021. Despite the optimal K = 1 obtained by sNMF, further partitioning of the genome revealed a clinal pattern of variation, with the northern populations of the central and Mediterranean transects carrying an important genomic component assigned to the orange cluster (0.65 for K = 2, Fig. 2A). This component decreased gradually towards the south and is absent in most Atlantic populations. Greater K values (K ≥ 3) highlight the distinctiveness of the Atlantic populations. The clinal pattern of variation in the Mediterranean (MT) and central populations (CT) is captured by PC2, with the distinct Atlantic populations (AT) captured by the PC1 generated by PCAdapt fast (Fig. 2B). These genome-wide results confirm the Iberian cline captured by the 383 SNPs, and the claim that modern beekeeping has not disrupted the natural variation pattern in IHBs^40^.

### Signatures of Local Adaptation

#### Genetic-Environment Associations (GEA)

To identify potential selective pressures driving local adaptation in the IHB, the GEA methods Samβada and LFMM were employed in the genome scan. A total of 38,683,470 univariate models (1,289,449 SNPs × 2 alleles × 15 environmental variables) were processed by Samβada. Over 1,305 SNPs were identified as outliers (false discovery rate, FDR < 0.05; Supplementary Table S2). The most frequently associated environmental variables were longitude (long; 1,071 models, 31%), precipitation in August (prec8; 758 models, 22%), May (prec5; 368 models, 11%), and January (prec1; 336 models, 10%). The 12 top-ranked models (Gscore >50) identified 6 SNPs, which were located in genes GB40077 (1 SNP), GB54460 (1 SNP), GB45499 (2 SNPs) and GB48105 (2 SNP). Of the six SNPs, three SNPs were non-synonymous, with the strongest (Gscore = 59.0) tagging GB40077 and the other two tagging GB45499 (see further details in the protein modelling section), two were located in introns in the immediate vicinity of exons (between 85 and 225 bp), and one was in a synonymous position (Supplementary Table S2). The SNPs marking GB40077, GB54460 and GB45499 were associated with longitude whereas the two SNPs located in gene GB48105 were associated with precipitation in January.

A total of 1,416 (FDR < 0.05), 360 (FDR < 0.02), and 220 SNPs (FDR < 0.01) were identified by the LFMM method (Supplementary Table S3 and Fig. S3). The strongest 21 SNPs (defined by a cut-off level of −log10(q-value) > 4) were located in introns (11 in GB46620, 3 in GB43005, 1 in GB4810, 1 in GB54460), 1 in UTRs (GB48105), and 3 in exons (1 non-synonymous in GB46620, 1 non-synonymous in GB40077, 1 synonymous in GB48105). A single SNP mapped to an intergenic region, although close to a gene (207 bp upstream of GB46621). Most SNPs were associated with latitude (11 in GB46620, 3 in GB43005, 1 in GB46621) and/or precipitation in May (12 in GB46620, 1 in GB46621). The variables precipitation in January and longitude were associated with only 3 (GB48105) and 2 (1 in GB40077, 1 in GB54460) SNPs, respectively.

**Figure 3:**
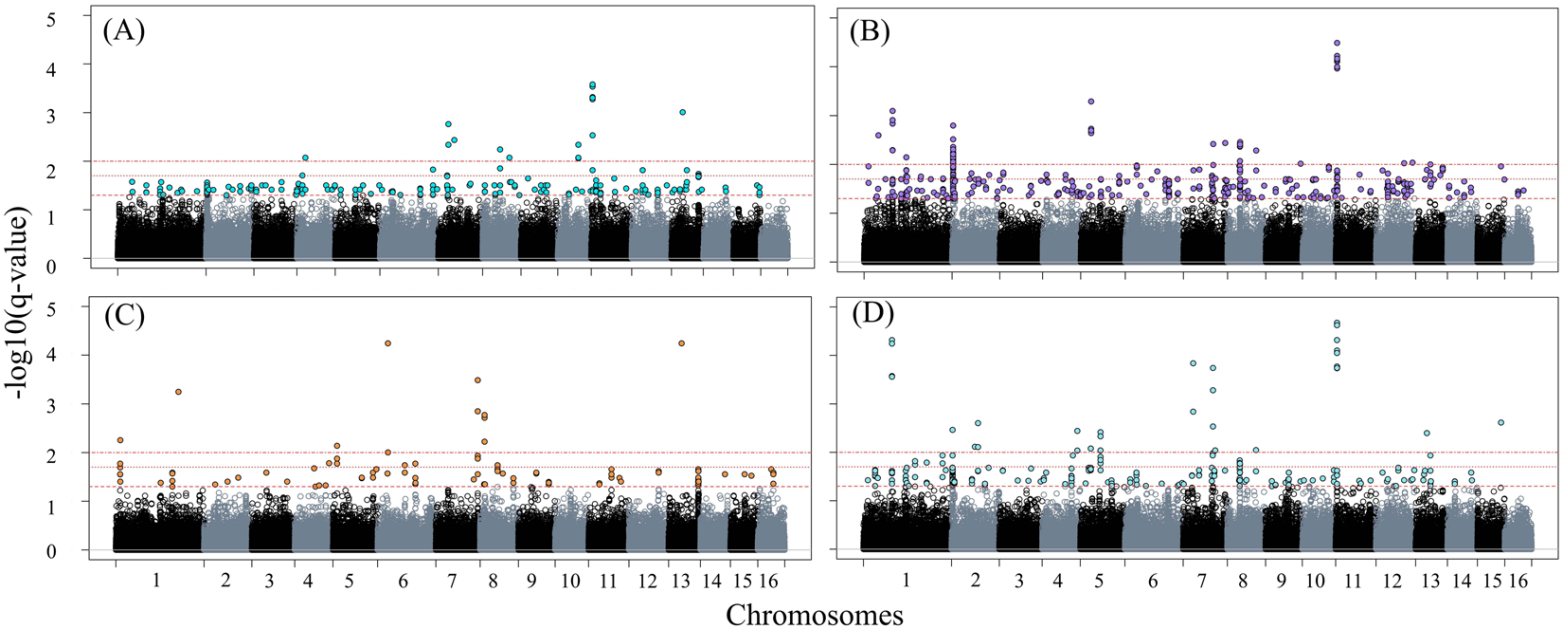
LFMM Manhattan plots. The plots represent the genome-wide distribution of significance values −log10 (q-value) obtained by LFMM for the environmental variables with the strongest associations. (**A**) precipitation in August: 351 SNPs, (**B**) precipitation in May: 596 SNPs, (**C**) longitude: 113 SNPs, (**D**) latitude: 385 SNPs. The red lines indicate FDR values of 0.05, 0.02 and 0.01.

A total of 598 SNPs overlapped between Samβada and LFMM (Supplementary Tables S4 and S5). These SNPs mapped to 126 genes and 99 intergenic regions. The variables precipitation in August and precipitation in May showed the greatest number of associated SNPs (227 and 152, respectively; Table 1, Fig. 3, Supplementary Tables S2 and S3), although longitude (113), latitude (102) and precipitation in January (101) were also predominant variables (Table 1). The variable latitude shared 52% of the SNPs with precipitation in August whereas longitude shared 35.40% of the SNPs with precipitation in January and 14.16% with cloud cover in April (cld4) (Supplementary Table S6).

**Table 1:** Environmental variables and number of associated SNPs identified exclusively by LFMM or Samβada and simultaneously by both methods.

Environmental variables	LFMM	Samβada	Overlapping
Precipitation August	124	152	227
Precipitation May	444	32	152
Longitude	0	423	113
Latitude	283	56	102
Precipitation January	63	67	101
Insolation April	18	55	50
Cloud cover April	165	8	32
Temperature min. January	58	27	18
Cloud cover July	24	4	10
Relative humidity January	27	21	6
Temperature min. June	1	16	6
Land cover	26	3	6
Relative humidity June	19	20	4
Relative humidity March	111	2	0
Altitude	0	3	0

There was an enrichment of SNPs detected by both GEA methods in exons (P-value < 2.20 × 10^−16^), UTRs (P-value = 8.8 × 10^−4^), introns <50 bp of exons (P-value = 8.01 × 10^−5^), and intergenic regions <1 Kb upstream of exons (P-value = 2.278 × 10^−5^, χ^2^ test).

#### PCAdapt fast

**Figure 4:**
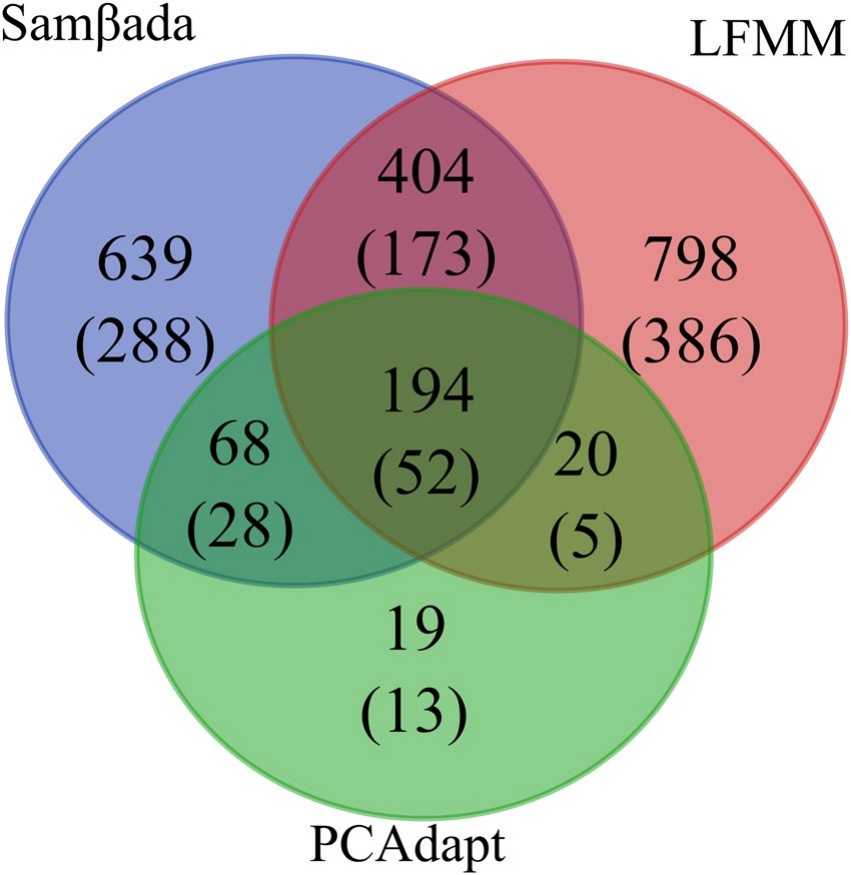
Overlapping SNPs identified by the three genome-scan methods. Numbers in the intersection regions represent overlapping SNPs among two or three methods. Numbers in parentheses show the corresponding genomic regions harbouring the SNPs.

For further cross-validating selection and reducing detection of spurious signals, we combined the GEA methods with the differentiation-based PCAdapt fast. A total of 285 outlier SNPs were identified by PCAdapt (FDR < 0.05; Supplementary Fig. S4 and Tables S5 and S7), of which 266 (93.3%) were cross-detected by the GEA methods (Fig. 4). From the 285 SNPs, 84 were located in 36 intergenic regions and 201 in 61 genes. The genes containing the highest number of SNPs were GB49881 (40), GB49882 (27), and GB46620 (18).

Putative targets of selection identified by PCAdapt fast were enriched in exons (P-value < 2.20 × 10^−16^, χ^2^ test), in intergenic regions <1 Kb upstream (P-value < 2.20 × 10^−16^, χ^2^ test) of exons, and in introns <50 bp of exons (P-value = 3.1 × 10^−8^, χ^2^ test).

### The Strongest Candidate SNPs

A total of 670 SNPs were detected by at least two selection methods, 11.3% were located in exons (41 non-synonymous and 35 synonymous SNPs), 3.0% in UTRs (20 SNPs), 46.1% in introns (309 SNPs, of which 28 were <50 bp of exons), 18.7% in intergenic regions adjacent to (2-2,000 bp; 125 SNPs, of which 42 were <1 Kb upstream) exons and 21% distant from (2,023–188,208 bp; 140 SNPs) exons.

The 670 SNPs exhibited |iHs| (integrated haplotype score) values ranging from 0.006 to 7.2 (Supplementary Tables S4 and S5). A total of 150 SNPs were strong candidates for recent ongoing selection as they showed a |iHs| > 2 (Supplementary Table S5). The two top-ranked SNPs displayed a |iHs| > 7, standing out by a remarkably strong selection signature. One of these two is located 834 bp upstream of the undescribed gene GB54883 and the other is a longitude-associated non-synonymous SNP located in GB55263 (see further details in the protein modelling section).

The great majority (405 SNPs, 60.4%) of the 670 SNPs were located in exons, introns and UTRs of 140 genes. Of these, 8 genes carried >10 SNPs (Supplementary Tables S4 and S5), mostly associated with precipitation in May and precipitation in August (Table 2). The aforementioned GB49881, GB49882, GB46620, and GB43005 are amongst the 8 genes and are highlighted by possessing 29, 19, 18 and 13 SNPs, respectively. Four genes were tagged by non-synonymous SNPs with GB48703 and GB48709 harbouring the most (Table 2).

**Table 2:** Candidate genes containing more than 10 SNPs detected concurrently by at least two selection methods. Genes marked in bold carry SNPs that were cross-detected by iHS and/or PCAdapt.

*A. mellifera* gene	# SNPs	SNPs distribution across genomic regions	Environmental variables
GB49881	29	29 Intronic	Long, prec1, cld4
**GB48698**	20	1 Exonic (syn), 16 intronic, 3 UTR	Prec8
GB49882	19	5 Exonic (syn), 14 intronic	Long, cld4
GB46620	18	2 Exonic (non-syn), 16 intronic	Lat, prec1, prec5, prec8, ins4
GB48709	13	3 Exonic (non-syn), 1 exonic (syn), 9 intronic	Lat, prec5, prec8
GB43005	13	13 Intronic	Lat, prec1, prec5, tmin1, tmin6, ins4
GB48703	12	3 Exonic (non-syn), 1 exonic (syn), 6 intronic, 2 UTR	Prec5, prec8
**GB48699**	11	1 Exonic (non-syn), 10 intronic	Prec8

The correlated environmental variables are longitude (long); latitude (lat); cloud cover in April (cld4); insolation in April (ins4); precipitation in January (prec1), May (prec5) and August (prec8); minimum temperature in January (tmin1) and (tmin6).

The highest stringency cross-validation based on the three methods (Samβada, LFMM and PCAdapt fast) identified 194 overlapping SNPs (Fig. 4 and Supplementary Tables S4 and S5). These were located in 39 genes, including 6 of the 8 genes containing >10 SNPs. From the 194 SNPs, 68 displayed elevated |iHs| values (>2.0) representing 14 genes and 11 intergenic regions (Table 3). The genes with the highest number of SNPs and the uppermost |iHs| values were GB49881 (28 SNPs, |iHs| > 3.0) and GB49882 (6 SNPs, |iHs| > 5.4). Interestingly, these genes share transcript sequence associated with the SHAW protein, for which there are five alternative variants, and are only 1,864 bp apart. This remarkably short intergenic region contained 15 SNPs detected by at least two methods with |iHs| > 1.7 (Supplementary Table S5).

**Table 3:** Genomic information, and associated environmental variables, of candidate genes cross-detected by Sam*β*ada, LFMM, PCAdapt and |iHs| > 2.

Gene	# SNPs	Genomic position	Environmental variables	Putative function
GB49881	28	Intronic	Long, prec1, cld4	Undescribed
GB49882	6	Intronic	Cld4	Sleep
GB49899	4	Intronic	Long, prec1	Pdz (post-synaptic density) domain
GB48696	4	Non-syn, syn, intergenic (<1235 bp)	Prec5, prec8	Inter-male aggressive behavior
GB48694	3	Intronic	Prec5	Undescribed
GB48105	2	Intronic (8 bp), UTR	Long, prec1	Neurogenesis
GB49874	2	Intergenic (<2060 bp)	Long, prec1	Undescribed
GB48702	2	Intronic (61 bp), intergenic (1, 111 bp)	Lat, prec5, prec8	Organism reproduction
GB51286	2	Intergenic (<17, 754 bp)	Long, prec1	Undescribed
GB48701	2	Intergenic (<804 bp)	Lat, prec5, prec8	Undescribed
GB48697	2	Syn, Intergenic (678 bp)	Lat, prec5, prec8	Undescribed
GB51427	1	Intergenic (952 pb)	Long	Response to fungus
GB49878	1	Intergenic (495 bp)	Long, prec1	Response to DDT
GB47226	1	Syn	Long, prec8	Undescribed
GB49879	1	Intronic (204 bp)	Long, prec1	Sleep
GB47281	1	Syn	Long, prec8	Ovarian nurse cell to oocyte transport;
GB47279	1	Non-syn	Long, prec8	Response to insecticide
GB48706	1	Intergenic (30 bp)	Prec5	Axoneme assembly
GB51401	1	Intergenic (180 bp)	Long	ATP-dependent RNA helicase activity
GB51396	1	Non-syn	Long	Oxidoreductase activity,
GB51422	1	Syn	Long	Undescribed
GB44109	1	Intergenic (2182 bp)	Prec1	Oxidation-reduction process

Putative functions were summarized from FLYBASE. The correlated environmental variables are longitude (long); cloud cover in April (cld4); precipitation in January (prec1), May (prec5) and August (prec8).

There was an enrichment of the 670 SNPs in exons (P-value < 2.20 × 10^−16^), UTRs (P-value = 0.0018), introns <50 bp of exons (P-value = 6.393 × 10^−6^), and intergenic regions <1 Kb upstream of exons (P-value = 2.73 × 10^−7^, χ^2^ test).

### Protein Modelling

**Figure 5:**
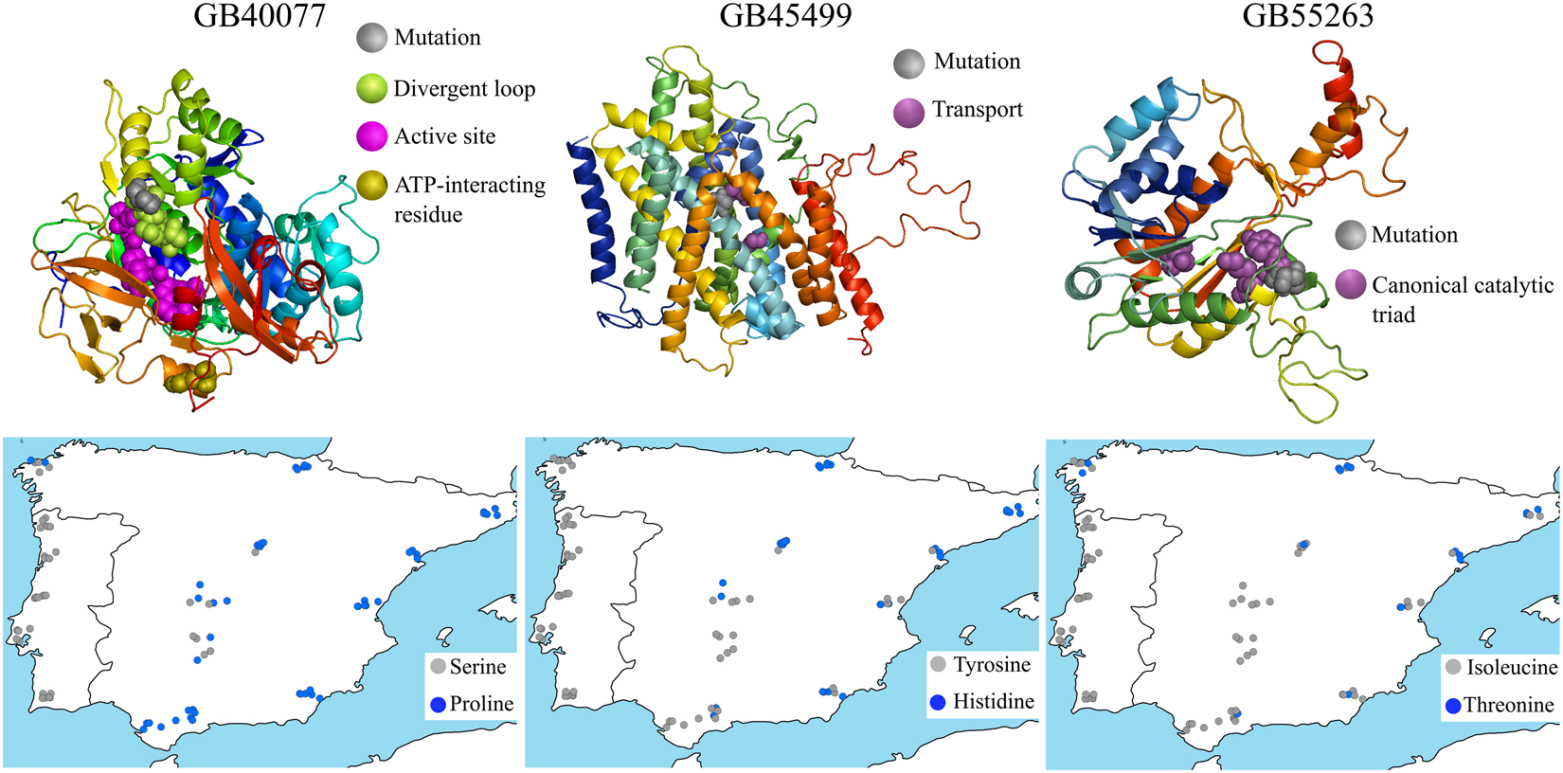
Predicted protein structures. The three genes harbour non-synonymous candidate SNPs, detected by three genome-wide methods, located nearby important places in the protein. The structures were predicted by Pymol considering the BeeBase reference amino acid sequences. The grey spheres represent the position and altered amino acids. The coloured spheres represent places with a known and important function in the protein. The maps depict the geographical patterns of the amino acids under selection.

To understand how SNPs causing amino acid changes could interfere with protein function, the 3D structure and stability were predicted for the different variants. A total of 41 non-synonymous SNPs were detected by at least two selection methods. The 41 SNPs were located in 29 genes. Protein prediction was available for only 11 of the 29 genes and 4 genes contained SNPs outside the 3D model (Supplementary Table S8). The remaining 7 genes (GB40077, GB45499, GB47279, GB48707, GB49875, GB51396, GB55263) were translated into a total of 37 protein variants (Supplementary Table S9). The gene GB49875 was the least diverse, with 3 variants, and gene GB40077 was the most diverse, with 9 variants (Fig. 5, Supplementary Fig. S5, Table S9). Most of these protein variants exhibited lower energy minimization than the reference (14 variants) and values of Gibbs-free energy (ΔΔG) > 0 (15 variants), with the highest ΔΔG values displayed by variants C and E of gene GB47279 (3.94 Kcal/mol and 2.29 Kcal/mol, respectively), indicating that the variants are less stable than the reference protein.

The non-synonymous SNPs tagging GB47279, GB48707, GB49875 and GB51396 produced amino acid changes outside of sites described as functionally important (see 3D structures in Supplementary Fig. S5). In contrast, GB40077, GB55263, and GB45499 contained non-synonymous SNPs that led to replacement of amino acids within or in the close vicinity of a functionally important site of the protein (Fig. 5). GB40077 and GB55263 encode proteins involved in lipid biosynthesis whereas GB45499 encodes a transport protein (Supplementary Table S4).

The single SNP detected inside the 3D prediction of GB40077 led to replacement of a proline (non-polar with restricted flexibility) by a serine (polar with low-flexibility) in position 363 (Fig. 5; Supplementary Table S9). GB55263 was also tagged by a single but strong SNP (|iHs| = 7.05; Supplementary Table S5), which causes a substitution of a threonine (polar with low flexibility) by an isoleucine (non-polar with moderate flexibility) at position 215 (Fig. 5; Supplementary Table S9). GB45499 was tagged by two non-synonymous SNPs. Of the two amino acid substitutions in this gene, only amino acid 74 was located inside the 3D prediction (Fig. 5). At position 74, the SNP leads to replacement of histidine (positive with moderate flexibility) by tyrosine (polar with moderate flexibility; Supplementary Table S9).

The geographical patterns exhibited by the variants of the amino acid under selection are shown in Fig. 5 and Supplementary Fig. S5. While variation of genes GB45499, GB55263, GB47279, GB48707, and GB51396 is oriented along a northeastern-southwestern axis, GB40077 and GB49875 display an eastern-western pattern with one the forms of the amino acid mostly confined to the Atlantic side of Iberia.

### Gene Ontology and Annotation

The power of the gene ontology (GO) analysis for uncovering the biological significance of candidate regions identified in whole-genome selection scans depends on the number of annotated genes available for the focal organism^51^. Of the 140 candidate genes identified here by at least two selection methods, only 109 were retrieved from the DAVID database. Hence, the GO analysis should be interpreted with caution as it may reflect a biased representation of candidate genes and miss biological functions. The 109 genes showed a significant enrichment (P-value < 0.05, before Bonferroni correction) for 6 functional terms (Supplementary Table S10), of which 4 formed one cluster (enrichment score = 2.58). The 4 terms were related with *membrane* of which only one (*integral component of membrane*) was significant after the Bonferroni correction. The remaining 2 functional terms (*olfactory learning* and *lucose/ribitol dehydrogenase*) were not clustered. While *olfactory learning* only included 2 genes, the fold enrichment was remarkably high (84.79).

The *membrane* cluster comprised 29 genes of which 16 could be grouped into three classes of proteins, including cell-surface receptors (7), transport (7), and cell-adhesion (1; Supplementary Tables S10 and S11). The cell-surface receptor genes (7 detected in GO analysis and GB45612 and GB48704) belong to four families, including the G-protein-coupled receptor family (GB40666, GB49166 GB48703 and its paralogue GB48704, GB49166 and GB51611), the ion-channel-coupled receptor (GB48639), the enzyme-coupled receptor (GB43446), and the CD36/scavenger receptor (GB49363). The transport proteins were represented by potassium channels (GB49879 and its paralogue GB49882) and transporters (GB45499, GB50262, GB49320, GB46597, GB53142, GB54678). Finally, the cell-adhesion proteins were represented by GB44159 and GB43719, being the latter undetected by the GO analysis (Supplementary Tables S4 and S10).

Although unrepresented in the GO enrichment analysis, many other genes are good candidates for local adaptation in the IHB as they are putatively implicated in the same biological function. These functions include reproduction with 7 genes, immunity with 11 genes, regulation of transcription with 7 genes, lipid storage and biosynthesis with 7 genes, olfaction with 8 genes, vision with 3 genes, and detoxification with 6 genes (Supplementary Table S11).

## Discussion

In this study we employed conceptually different analytical tools to disentangle signatures of selection from genome-wide geospatial variation in the IHB. By scanning 87 whole genomes, we were able to refine inferences previously made from a limited number of pre-ascertained biased SNPs^41^ and provide further insights into the molecular basis of local adaption in the IHB. In addition to providing unbiased information about the type of genes and biological processes putatively underlying local adaptation, we have never been so close to finding associated causal mutations.

The majority of the 41 non-synonymous outlier SNPs are likely causal mutations, especially those laying in genes GB40077, GB45499 and GB55263, as they could be linked to amino acid positions important for protein functioning^52–55^. On the other hand, it is possible that many outlier SNPs are hitchhiked with the actual genetic target of the selective event as 80% of the outlier SNPs were <5 Kb apart. Yet, the hypothesis that many linked multiple causal mutations have a functional role cannot be ruled out^56–58^.

A great proportion (88.7%) of cross-detected outlier SNPs are located in non-coding DNA, as opposed to the 11.3% exonic SNPs. A similar disproportionate fraction of non-coding to coding SNPs has been identified by whole-genome scans in other organisms, including humans^59^, fishes^56^, and fruit flies^23,60^. This finding together with the significant enrichment of (i) outlier SNPs laying in <1 Kb upstream from the transcription start site of 42 genes, where the promoter is expected to be located, (ii) intronic regions in the immediate vicinity of exons (<50 bp), and (iii) UTRs suggest that regulatory sequences are an important source of adaptive change in the IHB. Further identification of causal mutations is a challenging endeavour that will require more accurate and comprehensive annotations of the honeybee genome, and especially annotation of the non-coding regulatory DNA, along with evidence from biochemical and functional assays.

Support for selection is provided by functional annotations of candidate genes that can be directly related to colony fitness and it is particularly compelling when multiple candidate genes are implicated in the same biological function. While the GO enrichment analysis only detected 6 significant terms (4 related with *membrane*), functional annotations indicate that many fitness-related functions are represented by multiple genes (e. g. as reproduction with 7 genes or immunity with 11 genes). Other fitness-related biological functions were highlighted as they displayed strong selection signals. These include olfaction, circadian clock, and lipids biosynthesis and storage. Many of the candidate genes identified for the IHB were also detected by whole-genome selection scans for other honeybee subspecies^43,44,47^ (Supplementary Table S4), suggesting that they are adaptively important across diverse environments.

The GO analysis identified a cluster of 29 candidate genes encoding for membrane proteins in the IHB. The importance of membrane proteins in adaptation to new environments is evidenced by their rapid evolution compared with cytosolic proteins^61^. In this study, three candidate genes are highlighted in the group of membrane transport proteins. Gene GB45499 is one of strongest candidates for selection as it carries a mutation leading to an amino acid change in a site of the protein located in the transmembrane region and involved in transport activity^53^. The replaced amino acid is located in the alpha-helix and, together with two other amino acids, it is important to maintain the open pathway from the intracellular space^53^. Genes GB49879 and GB49882 are paralogous encoding voltage-gated K^+^ channels, which are putatively implicated in the circadian clock (see below). GB49879 was detected by all selection methods and exhibits a |iHS| = 2.19, indicative of strong signals of ongoing selection^62^. GB49882 is tagged by 19 SNPs, of which 5 are exonic. In addition to membrane transport protein, the selection scan identified three candidate genes in the group of membrane receptors all implicated in olfaction.

The adaptive relevance of olfaction is revealed by the significant enrichment of the GO term *olfactory learning* and identification of 8 candidate genes. GB48703 and GB48691 are amongst the most striking candidates deserving further investigation. GB48703 encodes an olfactory membrane receptor and carries 12 outlier SNPs, of which 3 are non-synonymous. GB48691 is implicated in olfactory learning and carries 9 outlier SNPs, of which one is non-synonymous. Unfortunately, protein prediction was not available for these genes hampering inferences on effects of the non-synonymous mutations in protein functioning. Colony fitness relies largely on olfactory perception. Olfaction is implicated in the learning process, which is crucial for the improvement of resources’ acquisition, as well as in a wide array of behaviours, including detection of possible dangers, recognition of potential mates, and social interactions^63,64^. Olfaction has also been shown to play a major role in the detection of brood cells infested by *Varroa destructor*^65^, an invasive mite that has been challenging honeybee health at unprecedented levels.

The honeybee relies on a circadian clock to synchronize foraging behaviour and reproductive swarming with the maximum daily and seasonal availability of food resources^66,67^. The importance of circadian rhythmicity in local adaption of IHBs is suggested by four candidate genes putatively operating in two functional components of the circadian clock: the oscillator and the output pathways. The core component “oscillator” is represented by GB52077. Its putative orthologue in *Drosophila* encodes for the transcription factor Period (*Per*). The honeybee *amPer* is an essential element of circadian rhythmicity, and its product is involved in a negative transcription/translational auto-regulatory feedback loop^68^. The development of strong circadian rhythms in honeybee foragers has been shown to be associated with changes in brain *Per* expression^67^. The component “output pathways” is represented by the striking candidates GB49879, GB49881 and GB49882. Genes GB49881 and GB49882 share transcript sequence associated with the SHAW voltage-gated K^+^ channel protein. GB49882 and GB49879 are paralogous encoding for SHAW and SHAW-like proteins, respectively. In *Drosophila*, the SHAW potassium channels regulate the intrinsic excitability in all neurons, being therefore important for output rhythms of the circadian clock^69^. The four clock genes were mostly marked by intronic outlier SNPs, suggesting that gene regulation is an important molecular mechanism to meet functional demands of circadian rhythmicity.

Seven lipid-related candidate genes mostly implicated in lipids biosynthesis and storage were detected in the IHB, being GB55263 and GB40077 amongst the top-ranked candidates possibly playing a central role in IHB adaptation. The non-synonymous SNPs mapped to these genes are likely causal mutations as they lead to replacement of amino acids located in functionally important sites of the proteins. The mutation in GB55263 leads to an amino acid replacement in a canonical catalytic triad^54^. The mutation in GB40077 leads to an amino acid replacement in a divergent loop^52^, which is important for mediating the protein-protein interaction or is part of the ATP binding site^55^. The *Drosophila* ortholog of GB40077 is implicated in lipid homeostasis^70^ and has been linked to the circadian clock^71,72^.

Precipitation in August, May and January are the variables most frequently and strongly associated with SNPs. While precipitation may act as a selective pressure by interfering with foraging, winter mortality, behaviour in the nest, and mating flights^73,74^, whether it is a direct cause of selection is unclear. It may very well be that precipitation operates indirectly by determining availability of pollen and nectar sources across space and time, which will not only influence foraging and colony build up but also reproduction. Due to the highly contrasting climates (e.g. average annual precipitation is 1336.3 mm in the northwest and 284.6 mm in the southeast), plant communities (wild plants or crops) and blooming seasons are very heterogeneous across Iberia^75^. This could favour evolution of locally adapted populations to food resources. An interesting example of such adaptation is provided by the existence of an ecotype of *A. m. mellifera* (the other M-lineage subspecies in Europe) that has an annual brood cycle fine-tuned with the phenology of an abundant floral source in the Landes region in France^76,77^.

Precipitation in August, May, and January covary with temperature, insolation, cloud cover, and precipitation in the other months. Multicollinearity may lead to incorrectly identifying a variable as causal when the true selective pressure is a correlated variable. However, it is also possible that selection is driven by composite environmental cues. For example, the mating behaviour of honeybee queens is influenced by a combination of temperature, wind, and cloud cover^73,74^.

Longitude and latitude showed a large number of associations (113 and 102, respectively). While longitude and latitude do not act directly on organisms, they serve as composite variables representing multiple environmental factors, any one or a combination of which could be exerting parallel selective forces. Latitude has been found to be associated with circadian clock genes in *Drosophila*^78–81^ and humans^82,83^, and now in honeybees. Clock genes are tagged by SNPs forming latitudinal and longitudinal gradients in Iberia. This finding suggests that circadian rhythmicity is involved in local adaptation in IHBs by matching important behaviours, such as feeding and reproduction, with the diverse daily and seasonal environmental oscillations of Iberia.

Using both genetic and environmental data, we identified candidate genes putatively under climate-driven adaptation. This information is particularly important in the context of rapid global climate change, helping us to understand the mechanisms employed by organisms to adapt to varying environmental conditions.

## Methods

### Sampling

A total of 87 haploid *A. m. iberiensis* males were collected in 2010 from 16 sampling sites distributed across three north-south transects: one along the Atlantic coast (AT: N = 31), one along the centre (CT: N = 33), and another along the Mediterranean coast (MT: N = 23; see Chávez-Galarza, *et al*.^41^ for further sampling details). The sites cover a wide variety of climates ranging from the semi-arid in the southeastern part of Iberia to oceanic in the northwestern part (Fig. 1). Each of the 87 individuals represents a single colony and apiary.

### Environmental Variables

Geographical coordinates, recorded for each apiary using a global positioning system (GPS), were used to obtain seven environmental variables from publicly available databases (WorldClim, Climatic Research Unit, OPENEI): precipitation (prec), minimum temperature (tmin), mean temperature (tmean), maximum temperature (tmax), cloud cover (cld), relative humidity (rh), and insolation (ins). These variables were integrated into a geographic information system (ArcGIS 9.3 from ESRI) to extract yearly, seasonal and monthly data. Arabic numerals appended to each environmental variable designate the month for which the variable was obtained; for example, prec5 refers to precipitation in May. In addition to climate, land cover was described for each apiary by calculating the percentage of level 3 land cover classes^84^ within a 3 km radius circular area (for further details, see Chávez-Galarza, *et al*.^41^).

To prevent potential problems caused by non-independency, environmental variables were first organized into orthogonal vectors by performing a principal component analysis (PCA) using the *ade4* package^85^. The strong correlation between many of the environmental variables in each vector means that they share a substantial amount of information and the relative importance of each variable is difficult to assess. Accordingly, variables that were correlated at |r| > 0.8^86^ were removed from the data set. From an initial set of 123 environmental variables, 13 uncorrelated variables together with longitude (long) and latitude (lat), which are proxies for climatic diversity, were retained for further analysis (Supplementary Tables S12 and S13 and Fig. S6). Each retained variable is representative of a group of highly correlated variables, as listed in the Supplementary Table S13. The two largest groups comprise 33 variables; one of them is precipitation in May, which represents a wide array of variables, including precipitation, temperature, cloud and insolation; the other group is minimum temperature in June, which only represents temperature. Latitude is correlated with 27 variables, most of which represent insolation (ins), but also spring and summer precipitation (Supplementary Table S13).

### Whole-Genome Sequencing and Filtering

Whole genome sequencing (WGS) was performed using the Illumina HiSeq 2500 platform, which produced a mean coverage of 11X, ranging from 3X to 23X (Supplementary Table S14). Sequencing libraries were generated using Illumina TruSeq^TM^ Sample Preparation kits. The 2 × 150 paired end sequence reads were mapped against the reference honeybee genome Amel_4.5 using the Burrows-Wheeler Aligner (BWA)^87^.

To improve the read mapping quality, PCR duplicates were identified and marked using Picard (http://broadinstitute.github.io/picard/) and realignment around indels was performed to correct inconsistently mapped reads using the Genome Analysis Toolkit (GATK)^88^. To facilitate parallelization, the reads were split per chromosome using SAMtools (http://samtools.sourceforge.net/) and the readgroups information was modified with Picard. Bayesian population-based SNP calling was implemented using FreeBayes^89^ across the 87 samples. To reduce poor mapping and spurious heterozygous positions, SNPs were removed that (1) had more than two alleles, (2) showed a quality score <50, (3) were present in less than 61 samples (70%), and (4) exhibited very high (>3000) or very low (<87) read depth (Supplementary Table S15). Haploid male data were intentionally misspecified to be diploid in the FreeBayes SNP calling process. Positions that showed more than 10 individuals as heterozygous were discarded, as they were unlikely to represent true SNPs. Missing genotypes were imputed by IMPUTE2^90^. SNPs showing a minor allele frequency (MAF) <0.05 were removed from the data set using PLINK^91^.

### Genomic Information

Annotation information was obtained for all SNPs, including physical position, strand orientation and SNP functional state (non-synonymous, synonymous, intron or exon UTR, or intergenic regions), using the reference genome Amel_4.5, the Official Gene Set 3.2 (BEEBASE), and the Entrez Gene of NCBI. To have a complete functional annotation of each candidate gene, putative Gene Ontology classifications were obtained based on homology to *Drosophila melanogaster* using FLYBASE. The sequence alignments spanned at least 50 peptides with an e-score of 0.5 to assign orthologs. Approximately 7,103 *D. melanogaster* genes were linked to honeybee orthologs using these criteria. DAVID v.8.0 (the Database for Annotation, Visualization and Integrated Discovery) was accessed to determine if candidate genes were enriched for a specific functional annotation^92^. Genes were considered as candidates for selection if they were tagged by one or more SNP outliers laying in exons, introns, or UTRs.

### Population Structure

Population structure was inferred from two different approaches: PCAdapt fast^7,93^ and sNMF^94^. PCAdapt fast infers population structure using latent factors or scores. The approach sNMF is based on sparse non-negative matrix factorization to estimate the genetic ancestry components for each individual^94^. Ten runs were performed in sNMF with *alpha* = 8 for each K value (1 to 10). Cross-entropy was used to guide the choice of the number of ancestral populations. To summarize and visualize the sNMF outputs, Q-plots were post-processed online with CLUMPAK^95^. The results from the PCAdapt fast and sNMF were used to create latent factors in models (see the section below for further details).

### Searches for Signatures of Local Adaptation

The whole genomes of the 87 IHBs were scanned for selection signals using three conceptually different methods (Samβada, LFMM and PCAdapt) and two data sets (a genomic data set and a combined genomic and environmental data set). The outlier SNPs detected by at least two methods were further examined using the haplotype-based method iHS and protein modelling for a secondary validation. Implementation of conceptually diverse approaches allows identification of potential false positives; by cross-validating outlier SNPs there is stronger evidence for selection^96,97^. These SNPs are the most promising candidates for biochemical and functional follow up studies.

The significance levels of Samβada, LFMM and PCAdapt were assessed using the false discovery rate (FDR) procedure^98,99^. To apply the FDR, the observed P-values should be uniformly distributed^100^. When this assumption was not met, we applied the empirical null-hypothesis technique to recalibrate the distribution^100^. Only SNPs with an FDR < 0.05 were considered as outliers.

### Genetic-Environment Association Methods

Two GEA methods were employed to search for signatures of local adaptation. One implements mixed models (LFMM) and the other a logistic regression model (Sam*β*ada). LFMM uses an MCMC algorithm for regression analysis that models random effects, such as population history and isolation-by-distance, as unobserved (latent) factors^101^. This approach has proven to be efficient in screening genomes for signatures of local adaptation, performing well in cases of weak selection, complex hierarchical structure and polygenic selection^13,97,102–104^. The program was run using 50,000 iterations and a burn-in of 25,000. Based on the ancestry estimates previously obtained with sNMF^94^ and PCAdapt^7^, two latent factors were assumed. Since LFMM uses a stochastic algorithm, five runs with different seeds were performed. To increase the power of the LFMM test, the median z-score and adjustment of P-value were calculated.

The other GEA method Sam*β*ada is a spatial approach that uses univariate logistic regression models to identify locus-environment associations and at the same time measures spatial autocorrelation^12,15^. Sam*β*ada was run for each of the 15 environmental variables. The analysis included global and local autocorrelation using a weighting factor based on the 25 nearest neighbours. The P-values were calculated from the Gscore.

### Frequency-Based Method – PCAdapt fast

The frequency-based PCAdapt fast approach^7,93^ implements a genome scan to detect genes involved in local adaptation by taking into consideration population structure. PCAdapt fast infers population structure using latent factors or scores, and searches for loci that are atypically related to population structure measured by factor analysis (h). To calculate the best K, PCAdapt fast was run with K = 10. Given that the best K was 2, as determined by eigenvalues, the software was run for the second time to infer the loci under selection for K = 2. The latent factors, which describe population structure, were plotted in the first two PCA components (PC1 and PC2).

### Haplotype-Based Method – iHS

The integrated haplotype score method, iHS, measures the strength of evidence for selection acting at or near a given SNP, tracking the decay of haplotype homozygosity for ancestral and derived haplotypes extending from a tested core^62,105^. To determine the SNP variants state (ancestral or derived), we performed a pairwise alignment between *Apis mellifera* (v4.5^106^) and *Apis cerana* reference genomes (v1.0^107^) using the default settings of SATSUMA^108^ whole-genome synteny package. Subsequently, the |iHS| values were estimated for candidate SNPs detected by at least two of the three previous methods using the Selscan package^105^ with default parameters: –max-extend 1,000,000 (maximum EHH extension in bp), –max-gap 200,000 (maximum gap allowed between two SNPs in bp), –cutoff 0.05 (EHH decay cutoff). The script NORM, provided by Selscan, was implemented to frequency-normalize the output using the default parameter–bins 100 (number of frequency bins) over all chromosomes. Values of |iHS| > 2 are indicative of strong signals of recent positive selection^62^.

### *In Silico* Analysis of 3D Protein Structure

Structures of related proteins were searched for on Phyre2^109^ and the SWISS-MODEL servers^110^. The five best matches were aligned and compared with a reference protein using MEGA7^111^; the structure with the best similarity and coverage was downloaded from the RCSB Protein Data Bank. The 3D structures of reference proteins and variants were modelled using SWISS-MODEL. FoldX^112^ and the 3Drefine^113^ servers were used to refine the 3D structures. Protein stability of each variant was predicted using the Gibbs-free energy (ΔΔG) calculated with the FoldX software. The minimum energy required for stable structure was estimated using GROMOS96 implemented in Swiss Pdb-viewer software^114^. Root-Median-Square Deviations (RMSD) between the reference protein and each variant were estimated using TM-score^115^. The 3D predicted protein structures were visualized in Pymol 0.99 (PyMOL Molecular Graphics System).

### Data accessibility

Sequence data of *A. m. iberiensis* would be deposited at the ENA (www.ebi.ac.uk/ena) after the manuscript is accepted for publication.

## Electronic supplementary material

Supplementary Information

Supplementary Tables
