# Supplementary material for: Whole genome SNP-associated signatures of local adaptation in honeybees of the Iberian Peninsula
Source: Sci Rep. 2018 Jul 24;8:11145. doi: 10.1038/s41598-018-29469-5 (PMC6057950; doi:10.1038/s41598-018-29469-5)
Supplement: Supplementary file 1 — Supplementary Information [file 41598_2018_29469_MOESM1_ESM.pdf]

Supplementary Information

Whole genome SNP-associated signatures of local adaptation in honeybees of the Iberian Peninsula

Dora Henriques[1,2], Andreas Wallberg[3], Julio Chávez-Galarza[1,4], J. Spencer Johnston[5], Matthew T. Webster[3], M. Alice Pinto[1*]

[1]Mountain Research Centre (CIMO), Polytechnic Institute of Bragança, Campus de Sta. Apolónia, 5300-253 Bragança, Portugal

[2]Centre of Molecular and Environmental Biology (CBMA), University of Minho, Campus de Gualtar, 4710-057 Braga, Portugal

[3]Department of Medical Biochemistry and Microbiology, Science for Life Laboratory, Uppsala University, SE -751 23 Uppsala, Sweden

[4]Instituto Nacional de Innovación Agraria (INIA), Av. La Molina 1981, La Molina, Lima, Peru

[5]Department of Entomology, Texas A&M University, College Station, TX 77843-2475, USA

Supplementary Figures

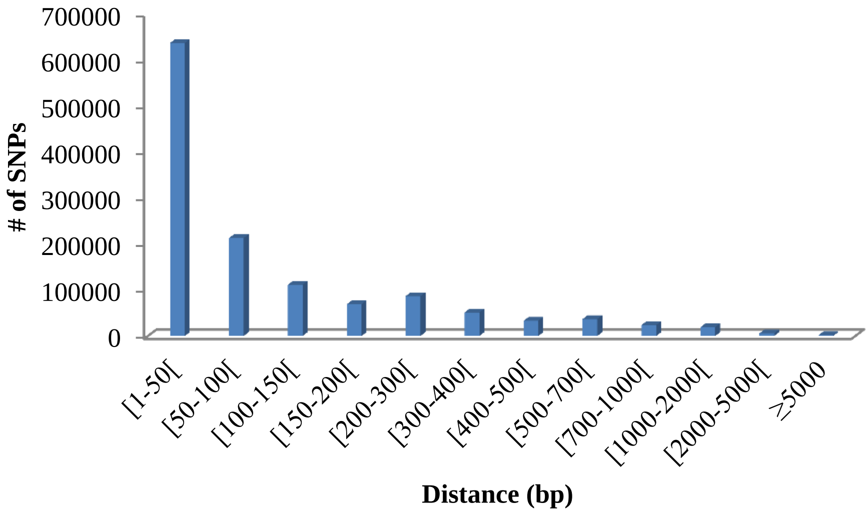

Supplementary Fig. S1. Distribution of the distance between the 1,289,449 SNPs across genomic regions. The average physical distance between SNPs was 170.3 bp varying between 1 bp and 136,266 bp.

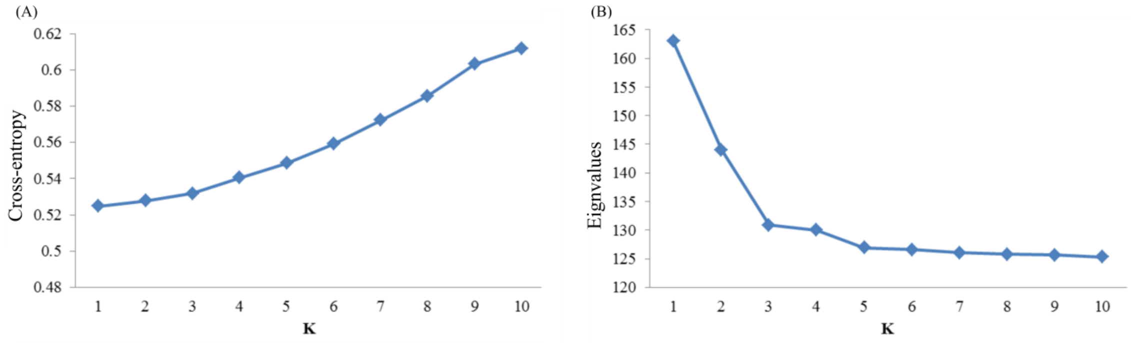

Supplementary Fig. S2. Graphical display of the two methods (cross-entropy and eigenvalue) used to predict the optimal K in the analysis of population structure.

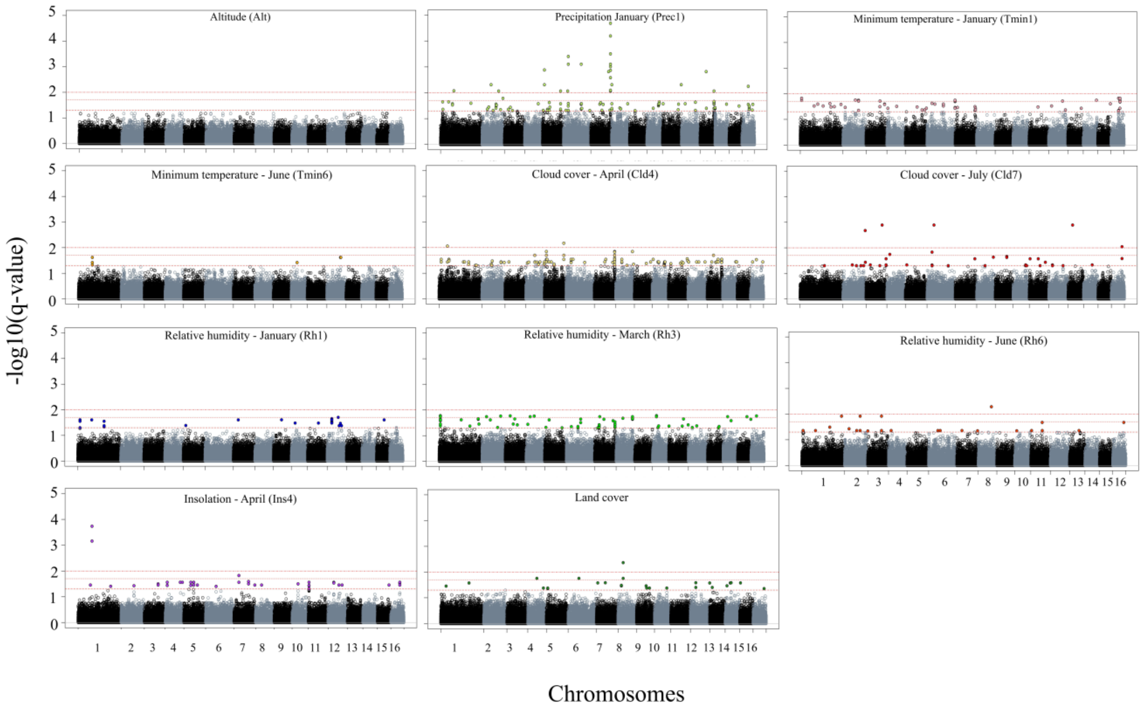

Supplementary Fig. S3. Manhattan plots representing the genome-wide distribution of significance values –log10 (q-value) obtained by the genetic-environment association approach LFMM for 11 environmental variables. The red lines indicate FDR values of 0.05, 0.02 and 0.01.

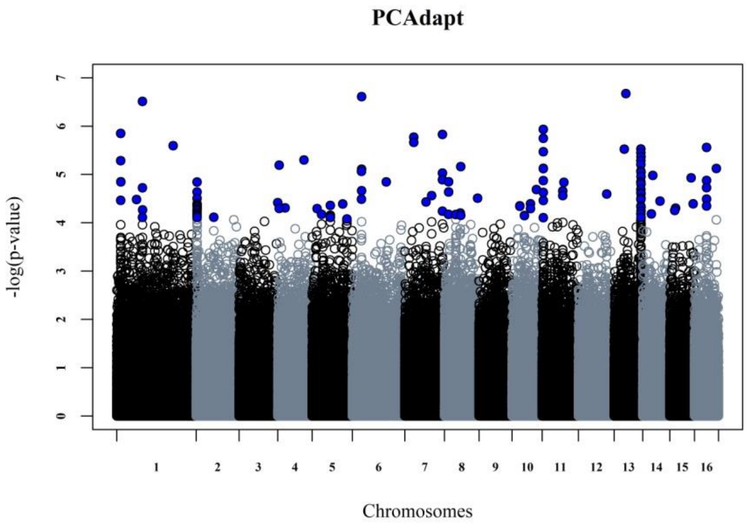

Supplementary Fig. S4. Manhattan plot representing the genome-wide distribution of significance values –log10 (P-value) obtained by PCAdapt fast. The blue dots represent the candidate SNPs.

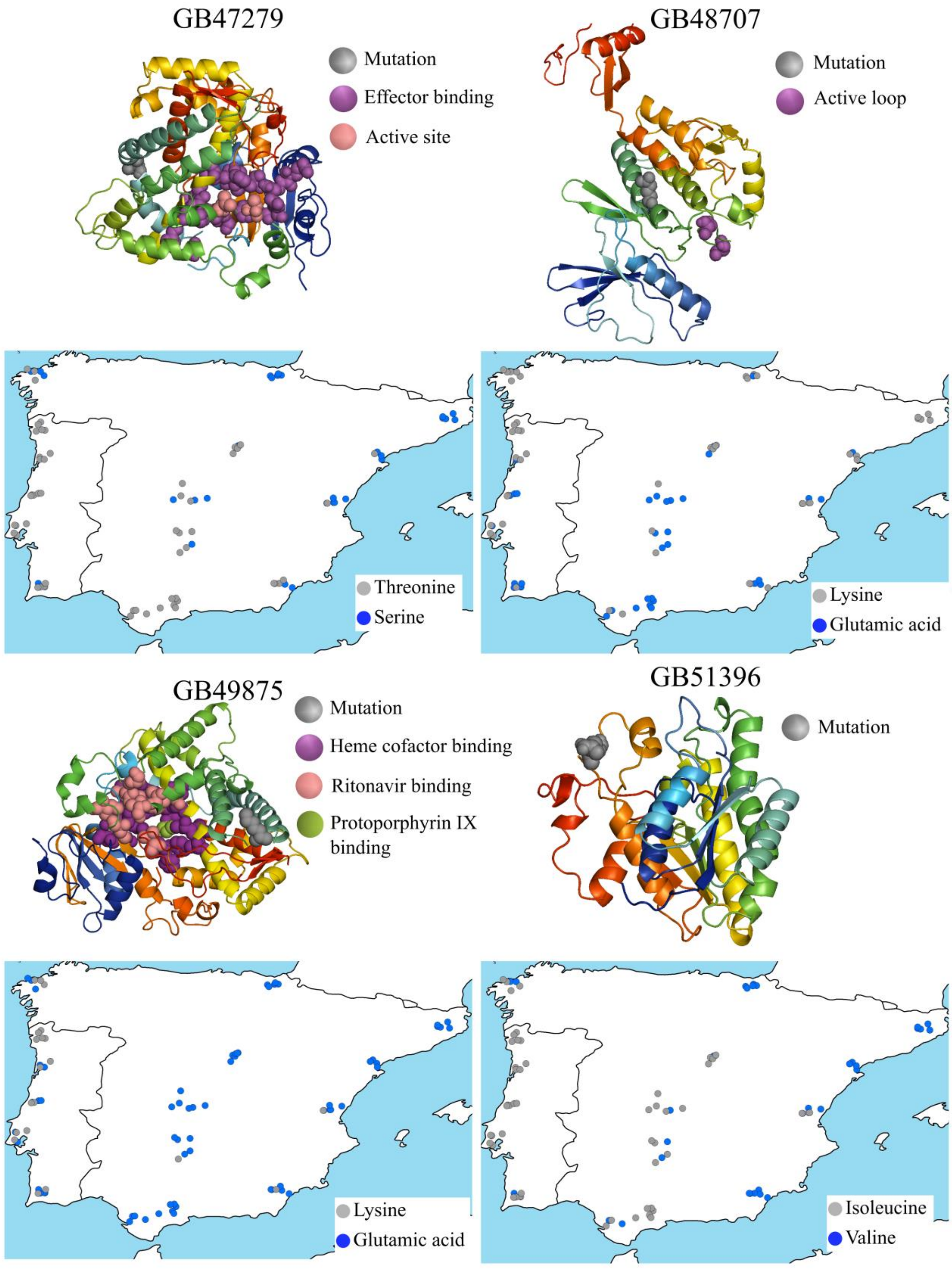

Supplementary Fig. S5 Predicted protein structures of four genes harbouring non-synonymous SNPs detected by three genome-wide selection methods. The structures were

predicted by Pymol considering the BeeBase reference amino acid sequences. The grey spheres represent the position and altered amino acids. The maps depict the geographical patterns of the amino acids under selection.

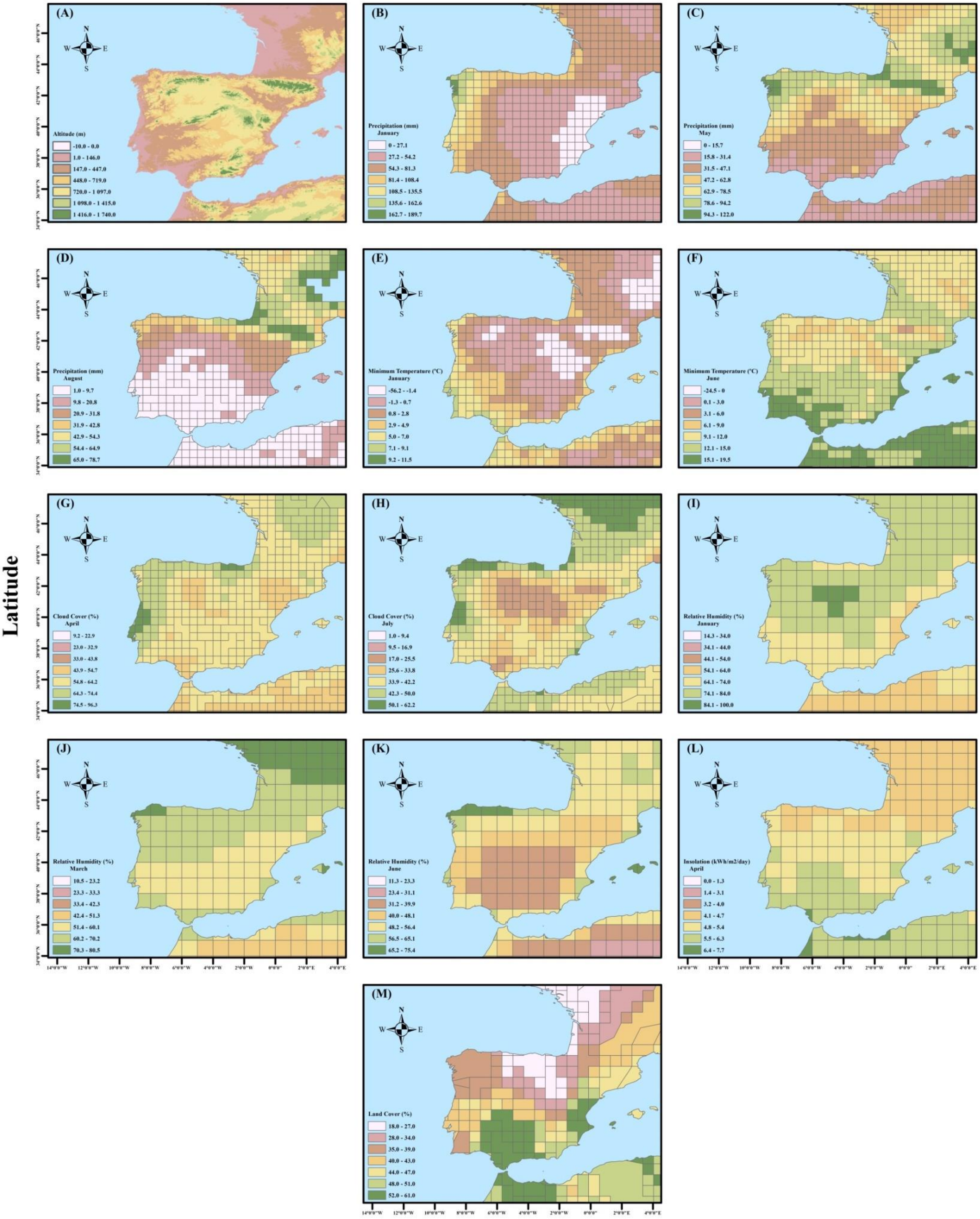

Latitude
(A)
N
W
E
S
Altitude (m)
-10.0 - 0.0
1.0 - 146.0
147.0 - 447.0
448.0 - 719.0
720.0 - 1 097.0
1 098.0 - 1 415.0
1 416.0 - 1 740.0
(B)
Precipitation (mm) January
0 - 27.1
27.2 - 54.2
54.3 - 81.3
81.4 - 108.4
108.5 - 135.5
135.6 - 162.6
162.7 - 189.7
(C)
Precipitation (mm) May
0 - 15.7
15.8 - 31.4
31.5 - 47.1
47.2 - 62.8
62.9 - 78.5
78.6 - 94.2
94.3 - 122.0
(D)
Precipitation (mm) August
1.0 - 9.7
9.8 - 20.8
20.9 - 31.8
31.9 - 42.8
42.9 - 54.3
54.4 - 64.9
65.0 - 78.7
(E)
Minimum Temperature (°C) January
-56.2 - -1.4
-1.3 - 0.7
0.8 - 2.8
2.9 - 4.9
5.0 - 7.0
7.1 - 9.1
9.2 - 11.5
(F)
Minimum Temperature (°C) June
-24.5 - 0
0.1 - 3.0
3.1 - 6.0
6.1 - 9.0
9.1 - 12.0
12.1 - 15.0
15.1 - 19.5
(G)
Cloud Cover (%) April
9.2 - 22.9
23.0 - 32.9
33.0 - 43.8
43.9 - 54.7
54.8 - 64.2
64.3 - 74.4
74.5 - 96.3
(H)
Cloud Cover (%) July
1.0 - 9.4
9.5 - 16.9
17.0 - 25.5
25.6 - 33.8
33.9 - 42.2
42.3 - 50.0
50.1 - 62.2
(I)
Relative Humidity (%) January
14.3 - 34.0
34.1 - 44.0
44.1 - 54.0
54.1 - 64.0
64.1 - 74.0
74.1 - 84.0
84.1 - 100.0
(J)
Relative Humidity (%) March
10.5 - 23.2
23.3 - 33.3
33.4 - 42.3
42.4 - 51.3
51.4 - 60.1
60.2 - 70.2
70.3 - 80.5
(K)
Relative Humidity (%) June
11.3 - 23.3
23.4 - 31.1
31.2 - 39.9
40.0 - 48.1
48.2 - 56.4
56.5 - 65.1
65.2 - 75.4
(L)
Insolation (kWh/m2/day) April
0.0 - 1.3
1.4 - 3.1
3.2 - 4.0
4.1 - 4.7
4.8 - 5.4
5.5 - 6.3
6.4 - 7.7
(M)
Land Cover (%)
18.0 - 27.0
28.0 - 34.0
35.0 - 39.0
40.0 - 43.0
44.0 - 47.0
48.0 - 51.0
52.0 - 61.0
14°0'0"W
12°0'0"W
10°0'0"W
8°0'0"W
6°0'0"W
4°0'0"W
2°0'0"W
0°0'0"
2°0'0"E
4°0'0"E

Supplementary Fig. S6. Maps showing the distribution of the 13 climatic variables used in the analyses. (A) altitude (alt), (B) precipitation in January (prec1), (C) precipitation in May (prec5), (D) precipitation in August (prec8), (E) minimum temperature in January (tmin1), (F) minimum temperature in June (tmin6), (G) cloud cover in April (cld4), (H) cloud cover in July (cld7), (I) relative humidity in January (rh1), (J) relative humidity in March (rh3), (K) relative humidity in June (rh6), (L) insolation in April (ins4), and (M) land cover.
